# Serum microRNA as a potential biomarker for the activity of thyroid eye disease

**DOI:** 10.1038/s41598-023-27483-w

**Published:** 2023-01-05

**Authors:** Namju Kim, Hokyung Choung, Yu Jeong Kim, Sang Earn Woo, Min Kyu Yang, Sang In Khwarg, Min Joung Lee

**Affiliations:** 1 Department of Ophthalmology, Seoul National University Bundang Hospital, Seongnam, Korea; 2 Department of Ophthalmology, Seoul Metropolitan Government-Seoul National University, Boramae Medical Center, Seoul, Korea; 3 Department of Ophthalmology, Seoul National University College of Medicine, Seoul, Korea; 4 Department of Ophthalmology, Seoul National University Hospital, Seoul, Korea; 5 Department of Ophthalmology, Asan Medical Center, Seoul, Korea; 6 Department of Ophthalmology, Hallym University College of Medicine, Hallym University Sacred Heart Hospital, 22, Gwanpyeong-Ro 170 Beon-Gil, Dongan-Gu, Anyang-Si, Gyeonggi-Do 14068 Republic of Korea

**Keywords:** Diseases, Endocrinology, Medical research, Molecular medicine

## Abstract

The aim of this study is to characterize the microRNA (miRNA) expression signatures in patients with thyroid eye disease (TED) and identify miRNA biomarkers of disease activity. Total RNA was isolated from the sera of patients with TED (n = 10) and healthy controls (HCs, n = 5) using the miRNeasy Serum/Plasma Kit. The NanoString assay was used for the comprehensive analysis of 798 miRNA expression profiles. Analysis of specific miRNA signatures, mRNA target pathway analysis, and network analysis were performed. Patients with TED were divided into two groups according to disease activity: active and inactive TED groups. Differentially expressed circulating miRNAs were identified and tested using quantitative reverse transcription-polymerase chain reaction (qRT-PCR) tests in the validation cohort. Among the 798 miRNAs analyzed, 173 differentially downregulated miRNAs were identified in TED patients compared to those in the HCs. Ten circulating miRNAs were differentially expressed between the active and inactive TED groups and regarded as candidate biomarkers for TED activity (one upregulated miRNA: miR-29c-3p; nine downregulated miRNAs: miR-4286, miR-941, miR-571, miR-129-2-3p, miR-484, miR-192-5p, miR-502-3p, miR-597-5p, and miR-296-3p). In the validation cohort, miR-484 and miR-192-5p showed significantly lower expression in the active TED group than in the inactive TED group. In conclusion, the expression levels of miR-484 and miR-192-5p differed significantly between the active and inactive TED groups, suggesting that these miRNAs could serve as circulating biomarkers of TED activity, however, these findings need to be validated in further studies.

## Introduction

Thyroid eye disease (TED) is the most common orbital inflammatory disease, and extrathyroidal manifestation of Grave’s disease (GD)^1^. Most patients with TED show distinct clinical features, including eyelid retraction, proptosis, extraocular muscle movement limitation, and inflammatory signs of eyelid and conjunctiva, which enable easy diagnosis. Although enormous progress has been made in elucidating the pathogenesis of TED, the exact mechanism of TED remains elusive, and therapeutic challenges and dilemmas remain.

Assessment of disease activity is crucial for designing treatment plans for patients with TED. Immunosuppressive agents, including corticosteroids, radiation treatment, and insulin-like growth factor-1 receptor (IGF-1R) antibodies are effective only in patients with active TED^2–4^. Surgical rehabilitation is the conventional treatment modality for inactive TED. The clinical activity score (CAS) is the most commonly used to measure the activity of TED in a clinical setting^5^. However, CAS contains subjective items such as spontaneous retrobulbar pain and pain on attempted eye movement. Furthermore, fine, quantitative analysis is not possible because each item of the CAS is binary^6^. These drawbacks warrant the development of novel biomarkers for measuring TED activity.

MicroRNAs (miRNAs) are a class of noncoding RNAs that post-transcriptionally regulate gene expression and are involved in a wide range of physiological and pathological processes^7^. In the past few decades, miRNAs have received increasing attention due to their roles in regulating gene expression. Deregulation of miRNAs results in mRNA dysfunction, leading to the development of pathologic conditions. Several studies have revealed a close association between miRNAs and a wide spectrum of diseases, including various cancers, diabetes, Alzheimer’s disease, and autoimmune diseases^8^. MiRNAs are secreted from various cell types into the extracellular space and then transported to the circulating blood, including peripheral blood^9^. Because miRNAs in body fluids are highly stable, these circulating molecules can be a good biomarker candidate that can be collected by a liquid biopsy^10,11^.

In the present study, we identified circulating miRNA expressions in patients with TED according to their activity to evaluate possible biomarkers for TED activity. We investigated differentially expressed miRNAs in patients with active and inactive TED. We further validated these differentially expressed circulating miRNAs using quantitative real-time PCR (qRT-PCR). We also aimed to identify enriched gene expression pathways of the differentially expressed miRNAs via Kyoto Encyclopedia of Genes and Genomes (KEGG) pathway analysis. This information may help enhance our understanding of the biological mechanisms underlying TED.

## Materials and methods

### Patients

This study adhered to the tenets of the Declaration of Helsinki, and the protocol was approved by the institutional review board of the participating centers. Written, informed consent was obtained from all participants. We recruited 15 participants who met the criteria for TED and healthy controls (HCs). The diagnosis of TED was made based on the characteristic clinical signs, including unilateral or bilateral eyelid retraction, eyelid swelling, proptosis, and diplopia related to restrictive strabismus^12^. Patients who had other ocular or systemic inflammatory diseases or who received systemic steroid or immunosuppressive agents within 6 months from the date of commencement of the study were excluded from this study. The activity of TED was assessed by the CAS consisting of seven items representing classic inflammatory symptoms and signs: (1) spontaneous pain behind the globe, (2) pain on attempted gazes, (3) swelling of the eyelids, (4) redness of the eyelids, (5) redness of the conjunctiva, (6) chemosis, and (7) swollen caruncle. Thus, the CAS ranged between 0 and 7, and a CAS ≥ 3 was considered an active TED.

### RNA extraction

Serum samples were isolated by centrifugation (1500 × *g*) from 10 mL of total blood and stored in a − 80 °C freezer until use. Total RNA was extracted from the serum of every sample using an miRNeasy Serum/Plasma Kit (Qiagen, Germantown, MD, USA) according to the manufacturer’s instructions^13^. RNA quality was measured using a NanoDrop 2000 spectrophotometer (Thermo Scientific™, Waltham, MA, USA).

### miRNA expression profiling

We analyzed the expression profiles of 798 miRNAs from serum samples using the NanoString nCounter Human v3 miRNA Panel (NanoString Technologies, Seattle, WA, USA) according to the manufacturer’s instructions. Data were analyzed and normalized using nSolver™ Software Analysis (NanoString Technologies, Seattle, WA, USA)^14^. NanoString data were used to obtain the miRNA expression in each sample as a fold-change value, and a fold change of ± 2 was considered significant. After background subtraction, the data were normalized to the geometric mean of the top 100 miRNAs. Paired Student’s *t* test and analysis of variance were used to calculate statistical significance.

### Bioinformatic analysis

The DNA intelligent analysis (DIANA)-miRPath online software suite was used to describe the potentially dysregulated pathways by up- and down-regulated miRNAs between active and inactive TED groups^15^. This software links miRNAs to target genes from Tarbase, v7.0^16^, and identifies the targeted KEGG pathways^17^. We used the “pathway union” option of the miRPath software. The p-values were obtained by Fisher’s exact test as an enrichment analysis method, and the false discovery rate (FDR) was estimated using the Benjamin and Hochberg method.

### Validation of miRNA profile

The relative quantities of selected miRNAs in serum samples were analyzed using the miRCURY LNA miRNA PCR Assay (Qiagen, Germantown, MD, USA)^18^. miRNA cDNA was amplified using the miRCURY LNA miRNA PCR primers as follows: hsa-miR-29c-3p, hsa-miR-4286, hsa-miR-941, hsa-miR-571, hsa-miR-192-5p, hsa-miR-502-3p, hsa-miR-597-5p, hsa-miR-296-3p, and Spike controls Cel-miR-39-3p (Qiagen, Germantown, MD, USA). Real-time PCR was performed on the StepOnePlusTM Real Time PCR System (Applied Biosystems, Waltham, MA, USA) using the miRCURY SYBR Green PCR Kit (Qiagen, Germantown, MD, USA), according to the manufacturer’s instructions. Thermal cycling conditions were 95 °C for 2 min, followed by 40 cycles of 95 °C for 10 s and 56 °C for 1 min. Quantification was carried out using StepOne software v2.2.2 (Applied Biosystems, Waltham, MA, USA). Relative miRNA expression levels were expressed as the ratio of the comparative cycle threshold (CT) to the expression levels of Cel-miR-39-3p (spike control). The 2^−ΔΔCt^ method was used to determine relative gene expression, and each reaction was performed in triplicate.

### Statistical analysis

All data are presented as the mean ± standard deviation (SD). Hierarchical clustering was performed on the samples using Euclidean distance and average linkage clustering to generate a heatmap and clusters for the samples^19^. The comparison of the differential expression of miRNAs between the two groups was performed using two independent sample t-tests or nonparametric tests. Receiver operating characteristic (ROC) curves were constructed, and the area under the ROC curve (AUC) was calculated to evaluate the discriminatory power of the candidate miRNAs. Two-sided p-values of < 0.05, were considered statistically significant. Statistical analyses were performed using SPSS software (version 21.0; IBM SPSS Statistics, Inc., Armonk, NY, USA) and GraphPad Prism version 7.0 (GraphPad Software, San Diego, CA, USA).

## Results

### Patient characteristics

The detailed clinical features of the 15 patients included in the discovery set are presented in Table 1. There were no significant differences in age or sex among the three groups. The duration of ocular symptoms was significantly shorter in the active TED group than in the inactive TED group (p = 0.0079). CAS and thyroid-stimulating immunoglobulin (TSI) were significantly higher in the active TED group than in the inactive TED group (p = 0.0079 and p = 0.0025, respectively).

**Table 1:** Detailed clinical and ophthalmologic characteristics of the discovery study population.

	Active TED (n = 5)	Inactive TED (n = 5)	Control (n = 5)	p-value
Age (mean, years)	46.4 ± 11.2	43.0 ± 16.6	39.6 ± 12.2	0.54
Female/Male	3/2	3/2	3/2	0.99
Duration of ocular symptom (months)	3.0 ± 1.87	28.0 ± 1.87	–	0.0079
Clinical activity score	0.4	4.4	–	0.0079
Thyroid stimulating immunoglobulin (SRR%)	568 ± 141	211 ± 118	–	0.0025

*TED* thyroid eye disease, *SRR* sample-reference ratio.

### Overall miRNA expression in patients with TED

We compared the miRNA profiles in the serum samples from 10 patients with TED and 5 healthy controls to identify dysregulated miRNAs using the NanoString nCounter miRNA panel. Using the screening criteria of significant p-value (< 0.05) and fold change FC < − 2 or > 2, 173 miRNAs were differentially downregulated in the serum of patients with TED versus HCs. Top-ranked miRNAs, which showed the largest fold change (FC < − 3.5), are listed in Table 2.

**Table 2:** List of the seven top-ranked miRNAs with significantly different serum levels in healthy controls and patients with thyroid eye disease.

Probe name	Control (n = 5)	Thyroid eye disease (n = 10)	Fold change	p-value
hsa-miR-302d-3p	29.75	3.95	− 7.54	0.02282071
hsa-miR-181b-2-3p	61.53	15.82	− 3.89	0.0108964
hsa-miR-4787-3p	21.32	5.5	− 3.88	0.03649701
hsa-miR-1304-5p	56.34	14.65	− 3.85	0.02528491
hsa-miR-660-3p	29.77	7.96	− 3.74	0.00470312
hsa-miR-4448	33.41	9.09	− 3.68	0.00563364
hsa-miR-146b-3p	12.89	3.66	− 3.52	0.01222381

### Discovery of candidate miRNAs for TED activity

**Figure 1:**
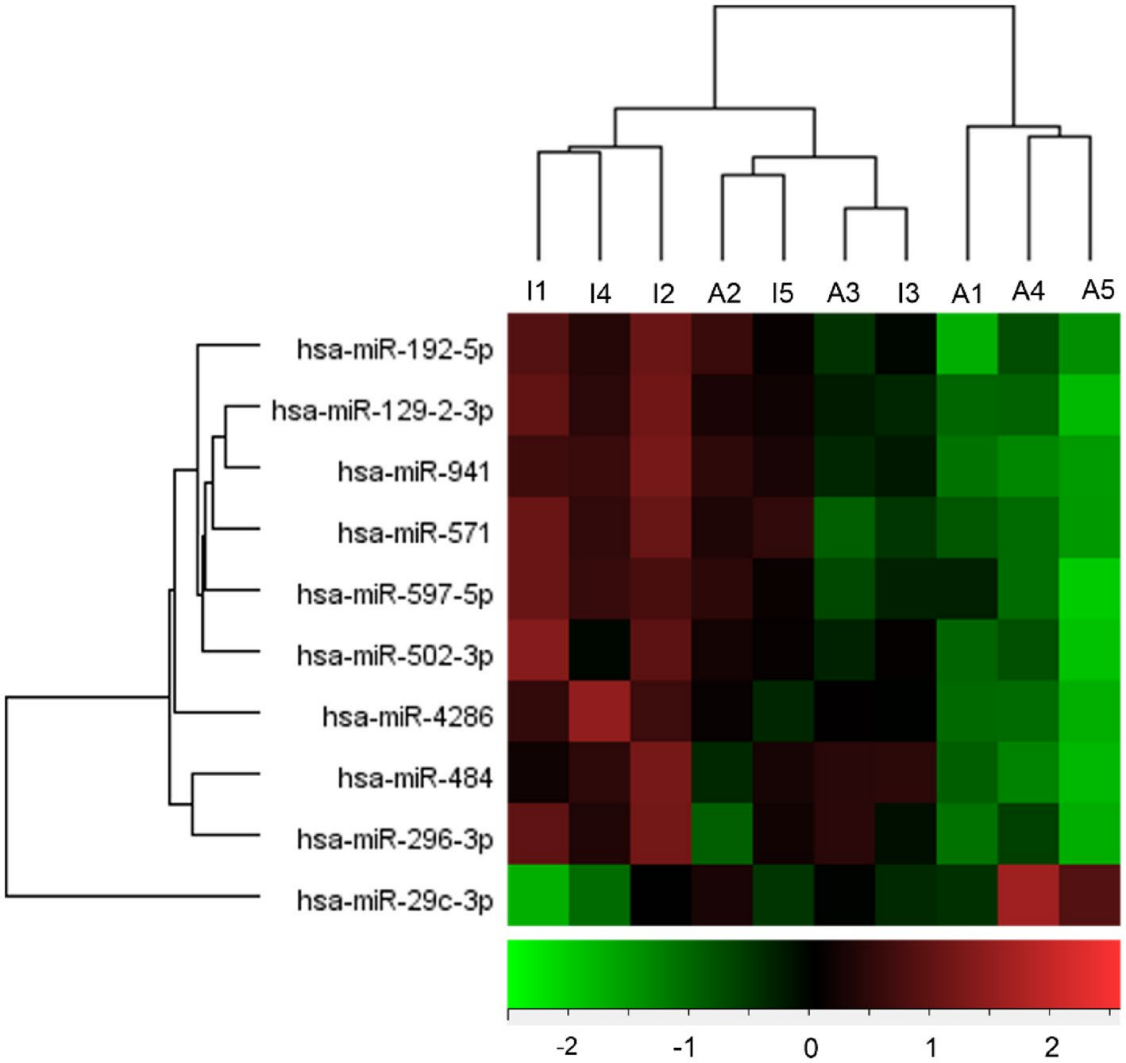
Differentially expressed microRNAs (miRNAs) (p < 0.05) were analyzed by hierarchical clustering of the values of the miRNA microarray signals. Red: upregulation; green: downregulation. The heatmap shows 10 differentially expressed miRNAs between active and inactive thyroid eye disease, using miRNA array data. *A* active thyroid eye disease group, *I* inactive thyroid eye disease group.

Patients with TED were subdivided into two groups according to the clinical activity: active and inactive TED groups. Among the 798 miRNAs analyzed, 10 candidate miRNAs were identified, which were significantly different between the active and inactive TED groups (Table 3). miR-29c-3p was significantly upregulated and nine miRNA probes were significantly downregulated (miR-4286, miR-941, miR-571, miR-129-2-3p, miR-484, miR-192-5p, miR-502-3p, miR-597-5p, and miR-296-3p) in the active TED group compared to the inactive TED group. A hierarchical clustering heatmap is shown in Fig. 1.

**Table 3:** Fold changes and associated p-values for 10 differentially expressed miRNAs between the active and inactive thyroid eye disease groups.

Probe name	Active TED (n = 5)	Inactive TED (n = 5)	Fold change	p-value
hsa-miR-29c-3p	76.34	31.84	2.4	0.03660034
hsa-miR-4286	19.97	43.6	− 2.18	0.03608293
hsa-miR-941	15.64	37.78	− 2.42	0.02348339
hsa-miR-571	13.69	33.95	− 2.48	0.01339567
hsa-miR-129–2-3p	13.74	37.77	− 2.75	0.02813069
hsa-miR-484	15.82	44.8	− 2.83	0.02365469
hsa-miR-192-5p	16.33	46.7	− 2.86	0.03596405
hsa-miR-502-3p	11.27	34.59	− 3.07	0.02692973
hsa-miR-597-5p	12.2	38.46	− 3.15	0.04822174
hsa-miR-296-3p	8.54	31.52	− 3.69	0.01912524

### Functional pathway analysis of candidate miRNAs

As a particular miRNA may act on several targets, the DIANA miRPath tool^15^ was used to identify KEGG pathways that were enriched in genes containing predicted target sites for the 10 differentially expressed miRNAs between active and inactive TED groups. Fisher’s exact test with a p-value threshold of 0.05 was used to determine which pathways were significantly targeted, and the results are shown in Table 4. According to the KEGG pathway maps, 12 pathways were significantly enriched, including fatty acid biosynthesis, ECM-receptor interaction, prion diseases, fatty acid metabolism, lysine degradation, adherens junction, steroid biosynthesis, viral carcinogenesis, cell cycle, proteoglycan in cancer, p53 signaling pathway, and focal adhesion.

**Table 4:** Enriched pathways: results using the DIANA-mirPath v3.0 software.

KEGG pathway maps	Enriched pathways	*Adjusted p-values
**Metabolism**		
Lipid metabolism	Fatty acid biosynthesis (hsa00061)	< 1 × 10^−325^
	Fatty acid metabolism (hsa01212)	7.77 × 10^−16^
	Steroid biosynthesis (hsa00100)	4.78 × 10^−5^
Amino acid metabolism	Lysine degradation (hsa00310)	6.38 × 10^−12^
**Environmental information processing**		
Signaling molecules and interaction	ECM-receptor interaction (hsa04512)	< 1 × 10^−325^
**Cellular processes**		
Cell growth and death	p53 signaling pathway (hsa04115)	0.0102
	Cell cycle (hsa04110)	0.0004
Cellular community	Focal adhesion (hsa04510)	0.0499
	Adherens junction (hsa04520)	3.25 × 10^−5^
**Human disease**		
Cancer: overview	Proteoglycans in cancer (hsa05205)	0.0075
	Viral carcinogenesis (hsa05203)	0.0006
Neurodegenerative disease	Prion disease (hsa05020)	< 1 × 10^−325^

*Adjusted p-values were obtained by using the Benjamini–Hochberg method.

### Validation of differentially expressed miRNAs using quantitative RT-PCR

**Figure 2:**
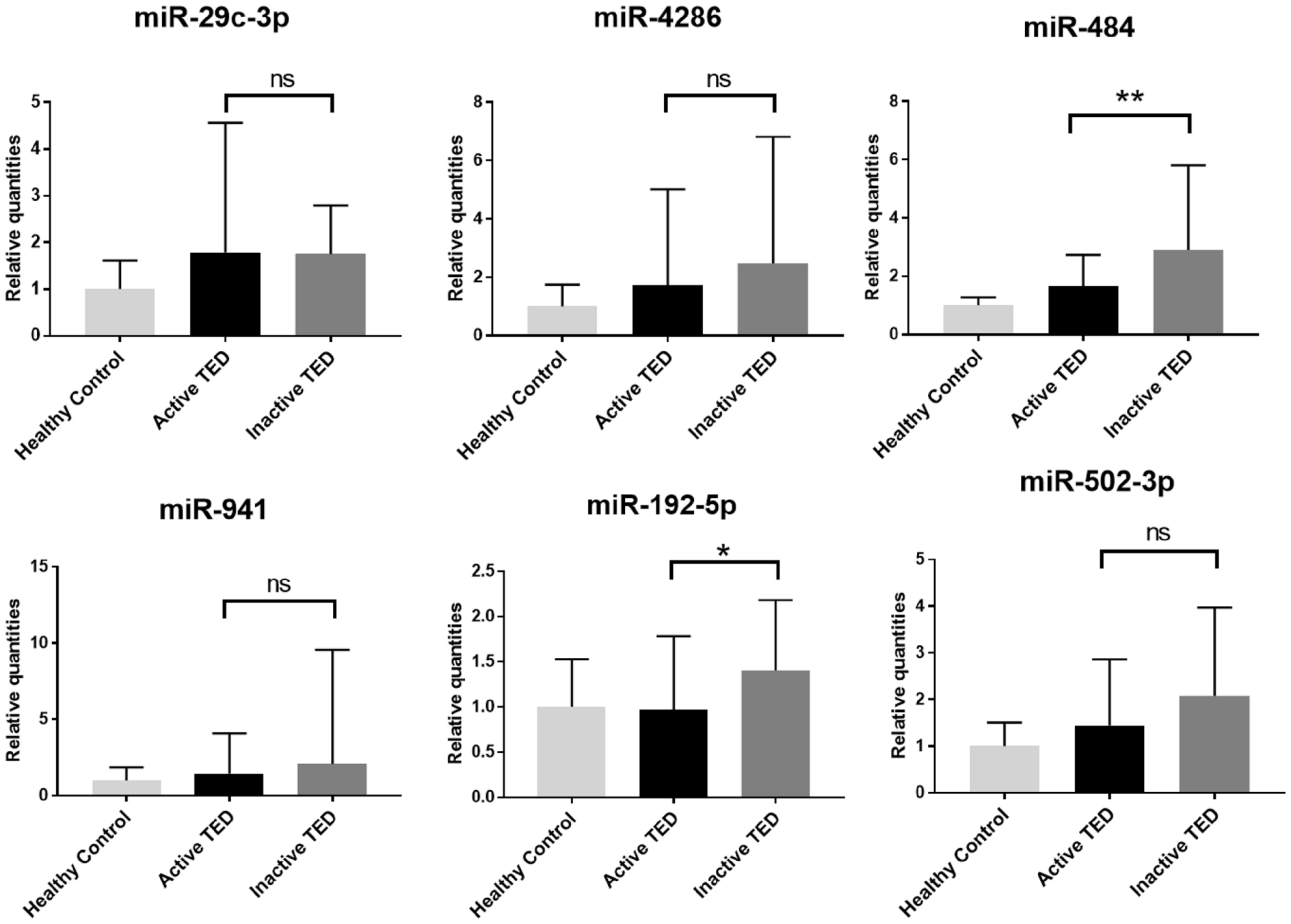
Analysis of serum samples from validation cohort using quantitative RT-PCR (healthy control (n = 4), active TED (n = 10), and inactive TED (n = 10), *p < 0.05, **p < 0.01).

**Figure 3:**
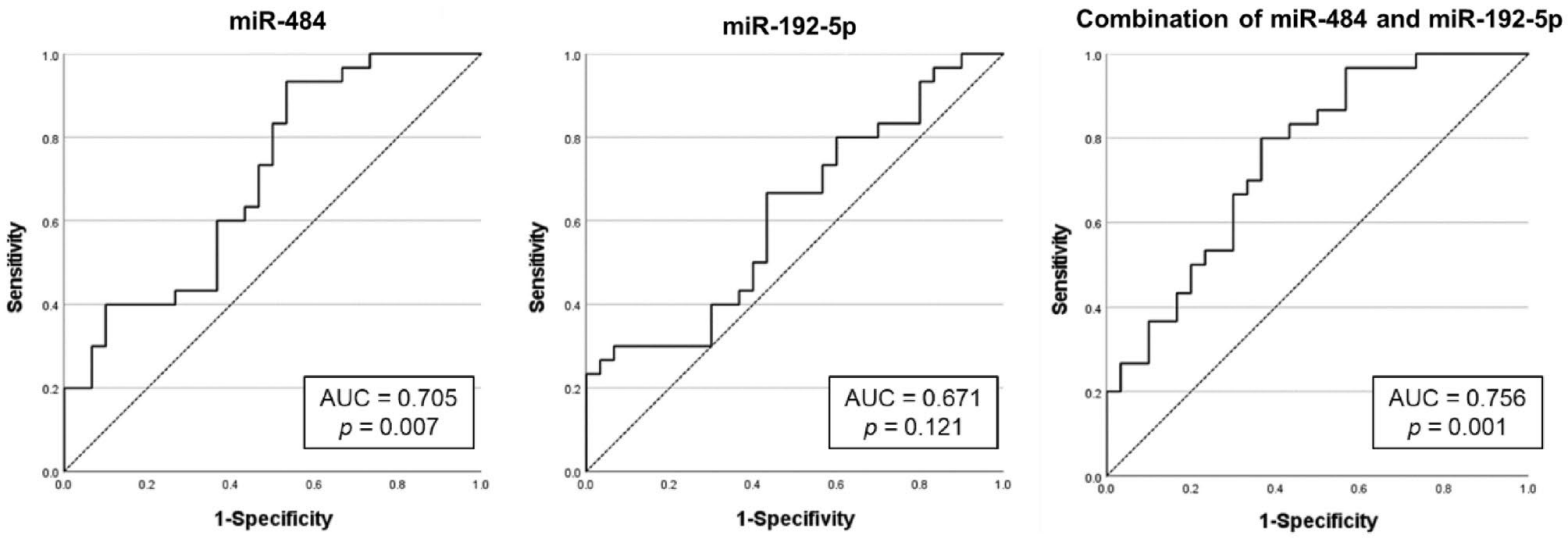
The receiver operating characteristic curve analysis of miR-484, miR-192-5p, and the combination of these two miRNAs to classify patients with active and inactive thyroid eye disease.

We performed qRT-PCR experiments to compare the levels of these differentially expressed miRNAs in a separate validation cohort (4 healthy controls, 10 patients with active TED and 10 patients with inactive TED). Six miRNAs (miR-29c-3p, miR-4286, miR-941, miR-484, miR-192-5p, and miR-502-3p) were successfully amplified, and the active TED group showed lower levels of miR-484 and miR-192-5p than the inactive TED group (Fig. 2). Subsequently, the discriminatory power of these two miRNAs to classify active and inactive TED was measured by ROC analysis. The ROC curve of miR-484 exhibited an AUC value of 0.705 (p = 0.007) indicating a good classifying performance; however, the ROC curve of miR-192-5p did not show a significant discriminatory performance, with an AUC value of 0.671 (p = 0.121). The combination of miR-484 and miR-192-5p showed better discriminatory power than each miRNA alone, with an AUC value of 0.756 (p = 0.001) (Fig. 3).

## Discussion

The present study aimed to investigate the potential role of circulating miRNAs as novel biomarkers of clinical activity in patients with TED and identified 10 miRNAs (miR-29c-3p, miR-4286, miR-941, miR-571, miR-129-2-3p, miR-484, miR-192-5p, miR-502-3p, miR-597-5p, and miR-296-3p) that were differentially expressed between active and inactive TED groups based on miRNA profiling. The KEGG pathway analysis revealed that most of the target genes of these miRNAs were involved in fatty acid biosynthesis, fatty acid metabolism, and ECM-receptor interaction, which have been closely linked with fat accumulation and inflammation. The miR-484 and miR-192-5p expression levels were found to be significantly lower in the active TED group than in the inactive TED group by validation with quantitative RT-PCR.

We profiled 798 miRNAs in serum samples from 10 patients with TED and 5 HCs using the NanoString nCounter system. In total, 173 miRNAs were differentially expressed in the serum of patients with TED as compared with HCs, and the majority of differentially expressed miRNAs in TED were downregulated. To the best of our knowledge, this is the first study to investigate the expression of circulating miRNAs using a wide panel of 798 miRNAs in patients with TED. We also used the NanoString nCounter system to investigate a microRNA profiling in serum samples from TED patients; this is known to be a highly sensitive method for detecting circulating miRNAs^20^ A large number of deregulated miRNAs suggest alterations in the biological processes involved in the pathogenesis of TED. When a fold change of FC < − 3.5 was applied, we identified 7 miRNAs that were most differentially expressed in patients with TED when compared with HCs (hsa-miR-302d-3p, hsa-miR-181b-2-3p, hsa-miR-4787-3p, hsa-miR-1304-5p, hsa-miR-660-3p, hsa-miR-4448, and hsa-miR-146b-3p). To date, few studies have focused on the role of miRNAs in the pathogenesis of TED. Some investigators have studied the function of miR-146a based on the putative pathogenesis of TED using an in vitro model of TED. Jang et al.^21,22^ investigated the level of miR-146a in cultured orbital fibroblasts and reported higher expression of miR-146a in orbital adipose tissues of patients with TED. The authors suggested that miR-146a plays a role in the regulation of inflammation and fibrosis in orbital fibroblasts. Tong et al.^23^ reported miR-21 acts as a mediator of TGF-ß1-induced collagen production.

We further analyzed miRNA profiles according to the activity of TED. Only 10 miRNAs showed significantly different expressions between the active and inactive subgroups of patients with TED. When these miRNAs were validated in separate samples by quantitative RT-PCR, we found that miR-484 and miR-192-5p were significantly decreased in the active TED group when compared with the inactive TED group. So far, there has been no study focus on investigating the definite role of miR-484 in autoimmune diseases, and the biological function of miR-484 in TED remains ambiguous. miR-484 has been reported to be associated with various cancers^24–26^. Interestingly, some studies have reported dysregulation of miR-484 in pulmonary fibrosis^27^ or hepatic fibrosis^28^. In addition, miR-484 was reported to be associated with fibrotic scar formation after spinal cord injury^29^. TGF-β mediates fibroblast distribution and fibrotic scar formation by activating miR-484 followed by ephrinB2 expression in fibroblasts. Regarding miR-192-5p, recent studies have demonstrated that it plays a role in several human diseases, including various cancers, asthma, arrhythmia, diabetes, and rheumatoid arthritis^30^. Although the functions of miR-192-5p are not fully explored, it is noteworthy that miR-192-5p plays a role in adipose differentiation and lipid homeostasis^31,32^. MiR-192-5p is also known to regulate diverse processes including oxidative stress, cellular proliferation, apoptosis, and inflammatory responses^30^. Interestingly, circulating miR-192-5p was also reported to be differentially expressed according to steroid responsiveness in patients with TED, supporting a possible role for this miRNA as a biomarker of TED activity^33^.

Despite making use of several laboratory tests, including recently developed serologic tests and imaging techniques, accurately assessing the activity of TED remains a challenge. CAS is widely accepted as the gold standard method for assessing TED activity^5,6,34^. The TSI assay has been reported to correlate with TED activity^35^. However, there is no standardized method for the detection of TSI and most techniques are intended for the qualitative detection of TSI, rather than a quantitative test. Regarding imaging techniques, various protocols have been studied, but no technique was found to be superior to CAS. More objective and quantitative molecular biomarkers based on pathogenic mechanisms would be beneficial for developing optimal treatment strategies for patients with TED.

Several studies have focused on the roles played by miRNA expression in TED. However, most of these studies were performed using in vitro models of TED and only a few existing studies have investigated serum miRNA levels in TED. Wei et al.^36^ investigated the association between the changes in levels of miR-146a and IL-7 with the clinical activity of TED using qRT-PCR and ELISA and reported lower miR-146a levels and higher IL-7 levels in active TED than in inactive TED. Shen et al.^33^ previously evaluated the level of circulating miRNAs according to the steroid responsiveness in TED patients. They used miScript PCR arrays for screening and qPCR for validation, like in case of the present study. However, they only included patients with active TED and compared the level of 84 miRNAs between steroid responders and non-responders. The strength of this study is that we performed the analysis of the expression of a wide panel of miRNAs. Here, we explored the levels of 798 miRNAs and used a very sensitive method to detect miRNAs in serum.

This study has some limitations. First, the sample size was limited; thus, the results from this study should ideally be validated in a much larger study population. Furthermore, the exact function of miR-484 and miR-192-5p in the pathogenesis of TED remains unclear, and in vitro or in vivo experimental studies regarding these miRNAs are warranted. Second, heterogeneous demographic, endocrinologic, and ophthalmologic factors can affect the expression of circulating miRNAs. Therefore, we plan to conduct further studies on the comparative analysis of miRNA expression in sequential serum samples collected from the same patient. Third, we performed only traditional biological experiments involving the screening of a broad panel of miRNAs, and validation of candidate miRNAs. Recently, there is a trend of developing computational models to identify the associations between miRNAs and various human diseases^37–39^. These computational models can effectively integrate heterogeneous biological data and predict potential associations between miRNAs and human diseases. Future studies are expected to develop a computational model that can predict miRNAs related to TED. Forth, we identified 10 miRNAs as potential biomarkers of TED activity using the NanoString nCounter system, but we successfully amplified only 6 of 10 miRNAs using qRT-PCR. We speculated that miRNAs that exist in scarce amounts in the serum might fail to be amplified by qRT-PCR, although they were detected using an amplification-free technique.

In conclusion, we investigated the expression of a wide panel of 798 miRNAs in patients with TED and identified 10 miRNAs which were differentially expressed between active and inactive TED. Validation studies revealed that the serum levels of miR-484 and miR-192-5p were significantly lower in active TED than in inactive TED. These miRNAs have the potential to serve as serum biomarkers of disease activity in TED. However, studies with larger sample sizes are needed to verify the roles of these miRNAs in the pathogenesis of TED.

## Data Availability

The datasets generated and analyzed during the present study are available in the Gene Expression Omnibus repository (GSE207995).
